# Long-Term Efficacy and Safety of Left Atrial Appendage Occlusion (LAAO) vs Direct Oral Anticoagulation (DOAC) in Patients with Atrial Fibrillation: A Systematic Review and Meta-Analysis

**DOI:** 10.31083/j.rcm2402044

**Published:** 2023-02-02

**Authors:** Aminah Abdul Razzack, Hassan Mehmood Lak, Greeshma Erasani, Sajedur Rahman, Nabeel Hussain, Bilal Farhat Ali, Srilatha Eapi, Farah Yasmin, Hala Najeeb, Ahmad Mustafa, Sanchit Chawla, Muhammad Bilal Munir, Amr F Barakat, Walid Saliba, Oussama Wazni, Ayman A. Hussein

**Affiliations:** ^1^Department of Internal Medicine, Dr. N.T.R University of Health Sciences, 520008 Vijayawada, India; ^2^Department of Internal Medicine, Cleveland Clinic Foundation, Cleveland, OH 44195, USA; ^3^Department of Internal Medicine, Jalalabad Ragib Rabeya Medical College and Hospital, 3030 Sylhet, Bangladesh; ^4^Department of Medicine, Saba University School of Medicine, JQG3+87 The Bottom, Netherland Antilles; ^5^Department of Medicine, Akhtar Saeed Medical and Dental College, 54000 Lahore, Pakistan,; ^6^Department of Medicine, Hackensack University Medical Center, Hackensack, NJ 07601, USA; ^7^Department of Internal Medicine, Dow University of Health Sciences, 74200 Karachi, Pakistan; ^8^Department of Internal Medicine, Staten Island University Hospital, Staten Island, NY 10305, USA; ^9^Section of Electrophysiology, Department of Cardiovascular Medicine, University of. California San Diego, San Diego, CA 92111, USA; ^10^Section of Electrophysiology, UPMC Heart and Vascular Institute, University of Pittsburgh, Pittsburgh, PA 15260, USA; ^11^Section of Cardiac Pacing and Electrophysiology, Robert and Suzanne Tomsich Department of Cardiovascular Medicine, Sydell and Arnold Miller Family Heart, Vascular and Thoracic Institute, Cleveland Clinic, Cleveland, OH 44195, USA

**Keywords:** left atrial appendage occlusion, atrial fibrillation, direct oral anticoagulation, ischemic stroke, bleeding

## Abstract

**Background:**

Prevention of stroke by anticoagulation is essential in patients with Atrial fibrillation (AF); with direct oral anticoagulants (DOACs) being preferred over warfarin in most patients. The Long-term efficacy and safety of DOACs vs. Left Atrial Appendage Occlusion (LAAO) remain unknown.

**Methods:**

Electronic databases (PubMed, Embase, Scopus) were searched from inception to February 10th, 2021. The primary endpoint was cardiovascular mortality. Secondary outcomes included incidence of ischemic stroke/transient ischemic attack (TIA) and systemicembolism. The safety endpoint was clinically relevant bleeding (a composite of major or minor clinically relevant bleeding).

**Results:**

A total of three studies with 3039 participants (LAAO = 1465; DOACs = 1574) were included. Mean age was 74.2 and 75.3 years in the LAAO and DOAC group respectively. Average follow-up period was 2 years. There was no difference in terms of cardiac mortality (RR 0.90, 95% CI 0.40–2.03; *p* = 0.81), ischemic stroke/TIA (RR 1.15, 95% CI 0.80–1.65; *p* = 0.46; $I^{2}$ = 0) and clinically significant bleeding (RR 0.77, 95% CI 0.50–1.17; *p* = 0.22; $I^{2}$ = 69) between the groups.

**Conclusions:**

Among patients with AF, LAAO was comparable to DOACs with similar efficacy and safety profiles.

## 1. Introduction

Atrial fibrillation (AF) is the most prevalent cardiac arrhythmia associated with increased mortality and impairment of quality of life [1]. There is a fivefold increased risk of stroke or systemic embolism (SSE), and 25% of all ischemic strokes reported in the elderly are a consequence of AF [2]. The mainstay therapy of anticoagulation for stroke prevention are direct oral anticoagulants (DOACs) and have primarily replaced Vitamin K antagonists (VKAs) due to a reduced risk of intracerebral bleeding [3]. However, bleeding rates of 2% to 3.6% have been reported in several randomized controlled trials (RCTs) [3]. In addition to drug compliance, bleeding remains an inherent problem with DOACs [4], underdosing and undertreatment [5]. Nonetheless, a significant number of patients are ineligible for anticoagulation with DOACs due to a history of high bleeding risk and intracranial hemorrhage [6]. Left atrial appendage occlusion (LAAO) devices have been developed primarily for patients that are contraindicated to oral anticoagulants. LAAO is a nonpharmacological stroke prevention strategy by which the left atrial appendage is closed and separated from the heart and circulation [7]. The main advantage of LAAO is decreased risk of bleeding by avoidance of long-term anticoagulation while still protecting from a stroke. Although not an alternative for DOACs, LAAO-related bleeding events are peri-procedural while OAC poses a life-long bleeding risk. Additionally, a study by Di Cori *et al*. [8] concluded that patients under monitored dosages of DOACs have a risk of developing LAA thrombi/emboli after transesophageal echocardiography. Although, this risk is deemed to be low but not negligible especially in patients with high risk predictors or with incorrect assumption of anticaoagulants [9]. Therefore, patients suitable for OAC therapy could also benefit from alternate procedures, such as LAAO which can play a role in non-inferior stroke prevention effect. The most widely used left atrial appendage occluders are the Watchman device and the Amplatzer Amulet [7]. In patients, LAAO has been reported to be a reasonable non-inferior alternative to warfarin for stroke prevention [10]; but the best strategy for stroke prevention risk remains unclear, and data comparing LAAO versus DOACs remains sparse. In order to assess the clinical implications of such stroke prevention strategies, we performed a meta-analysis of randomized controlled trials (RCTs) and observational studies to compare the pooled outcomes of LAAO and DOACs in patients suitable for OAC therapy, with a new onset nonvalvular AF.

## 2. Methods

### 2.1 Search Strategy and Selection 

We performed a systematic search of the online bibliographic databases PubMed, Embase, and Scopus and included relevant articles. The literature search was conducted from inception until the 10th of February 2021. A combination of keywords and MeSH terms, such as “atrial fibrillation”, “direct oral anticoagulants” “left atrial appendage occlusion”, “Watchman”, “Amplatzer”, “rivaroxaban”, “apixaban”, “edoxaban” and “dabigatran” were used to conduct a comprehensive search in the databases mentioned above. The reporting of the current review was in accordance with the Preferred Reporting Items for Systematic Reviews and Meta-Analyses [PRISMA] guidelines [11].

### 2.2 Inclusion Criteria

The inclusion criteria met the following specifications: (1) >10 AF patients were enrolled (2) Adult patients (age ≥18 years) who had paroxysmal, persistent, or permanent nonvalvular AF (3) Had undergone successful LAAO (4) Compared DOACs or Watchman/Amplatzer (5) Mean follow-up timewas ≥1 year (6) Provided data on cardiovascular outcomes, including the occurrence of stroke and adverse events of the procedure.

### 2.3 Exclusion Criteria 

Studies were excluded if individuals younger than 18 underwent Atrial Fibrillation ablation procedures. Also, if patients had comorbidities other than AF mandating anticoagulation, patent foramen ovale with large atrial septal aneurysm, mobile aortic plaque and symptomatic carotid arterial atherosclerosis. Studies with insufficient data, case reports, case series, conference abstracts meta-analyses, letters, editorials, and with less than ten patients (total n = 1504) were also excluded.

### 2.4 Data Extraction and Quality Assessment 

The screening of the article included extracting the study design, demographic characteristics, and various outcomes. Two authors (A.A.R and H.M.L). independently screened the titles and abstracts of all articles from the initial search. Any disparities concerning the assessment of the studies were rectified by the senior author. Screening also included searching reference lists of included studies (backward snowballing). For the quality assessment of included studies in the systematic review and meta-analysis, the Cochrane Risk of Bias tool for randomized controlled trials (RCTs) [12] and the Newcastle-Ottawa (NOS) scale for observational studies [13] was employed to ascertain the quality of studies by two independent reviewers (A.A.R and H.M.L).

### 2.5 Study Definitions and Endpoints

The primary outcome of interest was Cardiovascular Mortality. The secondary efficacy endpoints were (i) Ischemic stroke/Transient ischemic attack (TIA) and (ii) Systemic embolism. The safety endpoint was adjudicated clinically significant bleeding, a composite of major or minor bleeding. Procedure and device- related complications, including device-related deaths, device embolization, vascular complications, pericardial effusion, and other complications like malposition or leak, were also examined in the LAAO arm. Prior outcome definitions were not specified and were accepted as defined in the individual studies.

### 2.6 Statistical Analysis 

The Cochran-Mantel Haenszel method was used for statistical analyses. The random-effects model was used to calculate unadjusted risk ratios (RR) for the primary and secondary endpoints. The estimated effect size was reported as a point estimate and 95% confidence interval (CI). An alpha criterion of *p*-value ≤ 0.05 was considered statistically significant. The statistical model used was Higgins’s I- squared ($I^{2}$) assessment of study heterogeneity, with values <25%, 25–50%, 50–75%, and >75% corresponding to no, low, moderate, and high degrees of heterogeneity, respectively [14]. A confidence interval (CI) of 95% and a *p*-value < 0.05 were used in all our analyses to assess for statistical significance. The publication bias was depicted graphically and numerically as a forest plot and Egger’s regression test [15]. Statistical analyses were performed using the Cochrane review manager (RevMan) version 5.4 (The Cochrane Community, London, UK).

## 3. Results

The search strategy is shown in **Supplementary Fig. 1**. The initial screening yielded 5439 results. After the exclusion of duplicates, 2900 results were withheld for the screening of the title and abstract. Consequently, 1504 records were excluded due to ineligibility (reviews, editorials, non RCTs, ongoing trials, and abstracts). Finally, after screening 125 full-text articles, a total of 3 studies were included. These enrolled 3039 participants (1465 patients in the LAAO group and 1574 patients in DOAC group) [16, 17, 18]. The quality of the studies included was moderate. Amongst the three studies included, two were observational studies with matched cohorts, increasing the potential risk of selection bias [17, 18]. The two observational studies all had NOS scores >7 [17, 18]. Quality assessment findings of the included studies are summarized in **Supplementary Table 1, Supplementary Figs. 2,3**. 

### 3.1 Study Characteristics

Baseline demographics, comorbidities and study characteristics of studies included in the meta- analysis is summarized in Table 1 (Ref. [16, 17, 18]). The DOACs used in the RCTs were apixaban, rivaroxaban, dabigatran, and edoxaban. The anticoagulation strategy in the LAAO group was variable. The predominant antithrombotic therapy after LAAO was dual antiplatelet therapy (DAPT) with aspirin and clopidogrel for 1 to 3 months and then single antiplatelet therapy (SAPT) with aspirin for 6 to 12 months. The average follow-up period was two years. All studies reported ischemic stroke, cardiovascular death and all-cause mortality. For the safety endpoint, the DOACs trials reported clinically relevant bleeding, and LAA occluder trials reported both bleeding and device-/procedure-related complications. The mean age was 66.7 and 66.6 years in the LAAO and DOAC groups, respectively. 60.2% and 67.6% of patients were male in the LAAO and DOAC groups. The prevalence of hypertension in each group was 79.2% and 95.1% for LAAO and DOAC groups, respectively. 128 Prior MI was reported in 17.1% and 23.3% of the LAAO and DOAC groups. The prevalence of diabetes mellitus in each group were 30.1% and 38.1% for LAAO and DOAC arms, respectively. The left atrial appendage occluders that were used were Watchman device (Boston Scientific, Marlborough, Massachusetts, USA) and Amplatzer Amulet (Abbott Vascular, Santa Clara, California, USA).

**Table 1.** **Baseline demographics, comorbidities and study characteristics of studies included in the meta-analysis**.

Variable		PRAGUE 2020 [16]	Godino 2020 [17]	Nielsen-Kudsk 2021 [18]
Sample (n) LAAO/ DOAC		201/201	193/189	1184/1071
	Age (Mean ± SD)	73.4 ± 6.7/73.2 ± 7.2	74.2 ± 7.7/77.7 ± 6.9	75.1 ± 8.5/75.1 ± 10.5
	Male (%)	134 (66.7)/130 (64.7)	130 (54)/131 (78)	687 (64.2)/727 (61.4)
	CHA2DS2-VASc score (mean ± SD)	4.7 ± 1.5/4.7 ± 1.5	4.3 ± 1.5/4.8 ± 1.5	4.2 ± 1.6/4.3 ± 1.7
	HAS-BLED score (mean ± SD)	3.1 ± 0.9/3.0 ± 1.9	4.2 ± 1.0/3.3 ± 0.5	3.3 ± 1.0/3.4 ± 1.2
CAD risk factors				
	Hypertension	186/186 (100%)	169 (87.6%)/180 (95.2%)	896 (75.7%)/1023 (95.5%)
	Diabetes Mellitus	73 (36.3%)/90 (44.8%)	69 (35.8%)/43 (22.8%)	333 (28.1%)/424 (39.6%)
	History of Stroke	66 (32.8%)/63 (38.3%)	56 (29%)/55 (29.1%)	333 (28.1%)/376 (35.1%)
	Heart failure	88 (39.8%)/90 (44.8%)	NA	178 (15.0%)/223 (20.8%)
	Prior MI	30 (14.9%)/39 (19.4%)	37 (19.2%)/52 (27.5%)	NA
	Prior Bleeding	NA	133 (68.9%)/66 (34.9%)	794 (67.1%)/889 (83%)
	Year	2020	2020	2021
	Study Design	RCT	Observational PSM	Observational PSM
	Center	Multi-center	Single center	Multi-center
	Comparison Group	LAAC vs DOAC	LAAC vs DOAC	LAAC vs DOAC
	Sample size	402	382	2255
	LAAC Company	Amulet or Watchman or Watchman-FLX	Watchman™, Amplazer™	Amplatzer Amulet device
	Primary Endpoint	A composite outcome of stroke, transient ischemic attack, systemic embolism, cardiovascular death, major or non major clinically relevant bleeding, or procedure-/device related complications	Primary safety endpoint - Major bleeding, defined according to International Society of Thrombosis and Haemostasis (ISTH) classification. Primary Efficacy Endpoint = Thromboembolic events including ischaemic stroke, transient ischaemic attack (TIA), systemic embolism (SE), acute myocardial infarction (AMI)	A composite of ischemic stroke, major bleeding (Bleeding Academic Research Consortium), or all-cause mortality
	Secondary	Stroke (ischemic or hemorrhagic) or TIA, Systemic embolism, Clinically significant bleeding, Cardiovascular death; or Significant peri-procedural or device-related complications	Stroke, TIA, AMI, Systemic Embolism,as well as all bleedings, intracranial bleedings, gastrointestinal bleedings, overall death and cardiac death	Comprised each individual outcome of the primary composite outcome along with cardiovascular mortality, hemorrhagic stroke, and discontinuation of DOAC
	Bleeding Definition	Clinically significant bleeding was a composite of NMCRB, according to the ISTH criteria. Major bleeding includes either a decrease in hemoglobin of ≥2.0 g/dL during a 24-h period, transfusion of ≥2 units of packed red cells, bleeding at a critical site (intracranial, intraspinal, intraocular, pericardial, intramuscular with compartment syndrome, or retroperitoneal), or fatal bleeding. NMCRB is defined as bleeding requiring hospitalization or an invasive procedure but not meeting ISTH major criteria	Major bleeding, ISTH classification: decrease in the haemoglobin level of at least 2 g/dL, transfusion of at least two units of packed red blood cells, occurring at a critical site or resulting in death	Major bleeding (Bleeding Academic Research Consortium >3)
	Follow-up Duration	19.9 months	24 months	24 months
	Results	LAAC was noninferior to DOAC in preventing major AF-related cardiovascular, neurological, and bleeding events.	LAAO and DOACs performed similarly in terms of thromboembolic and major bleeding events up to two-year follow-up	LAAO in comparison with DOACs may have similar stroke prevention efficacy but lower risk of major bleeding and mortality.

*LAAC, Left Atrial Appendage Closure; DOAC, Direct Oral Anticoagulants; NMCRB, major and nonmajor clinically relevant bleeding; ISTH, International Society on Thrombosis and Hemostasis.

### 3.2 Clinical Outcomes

#### 3.2.1 Primary Efficacy Endpoint-Cardiac Mortality

**Fig. 1.**
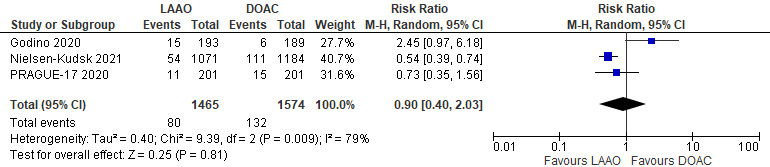
**Forrest plot comparing LAAO to DOAC for the primary efficacy endpoint of cardiovascular mortality**.

All three studies assessed cardiovascular death [14, 15, 16] and thus were eligible to be included in the meta-analysis. A total of 80 of 1465 (5.4%) deaths occurred due to cardiovascular causes in patients who underwent LAAO and 132 of 1574 patients (8.3%) in patients who were on DOACs. There was no statistically significant difference between the two groups regarding cardiovascular mortality (RR 0.90; 95% CI 0.40–2.03; *p* = 0.81) [Fig. 1]. However, there was moderately high heterogeneity among the studies included in the analysis ($I^{2}$ = 79%). For the sensitivity analysis, we tested if the removal of the study by Godino *et al*. [17] would lead to changes in the RR and significance. After excluding this study, the results suggested that the risk of cardiac mortality was higher in the DOAC group (RR: 0.56; 95% CI: 0.42–0.75; *p* = 0.0001), with homogenous findings ($I^{2}$ = 0%) (**Supplementary Fig. 4**).

#### 3.2.2 Secondary Efficacy Endpoints

**Fig. 2.**
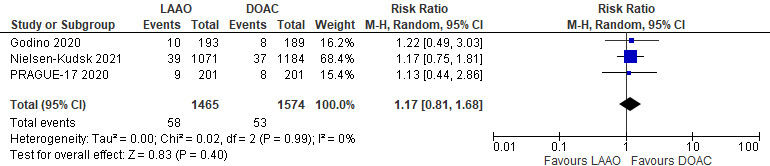
**Forrest plot comparing LAAO to DOAC for the incidence of Ischemicstrokes/TIA**.

*(i) Ischemic stroke/Transient ischemic attack (TIA): *All three studies presented data on risk of Ischemic stroke/TIA among the LAAO and DOAC groups. The incidence of ischemic strokes/TIA was comparable between the two groups. A total of 58 and 53 events occurred in the LAAO and DOAC groups, respectively. There was no significant difference between the two groups (RR 1.17; 95% CI 0.81–1.68; *p* = 0.40) with homogenous findings ($I^{2}$ = 0%) [Fig. 2].

**Fig. 3.**
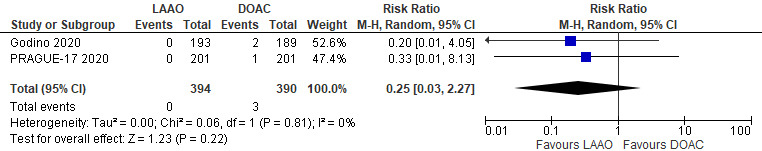
**Forrest plot comparing LAAO to DOAC for the incidence of systemic embolism**.

*(ii) Systemic Embolism (SE): *Two out of 3 studies evaluated the incidence of systemic embolism. When considering Systemic Embolism (SE) alone, no statistically significant difference was evident (RR 0.25; 95% CI 0.03–2.27; *p* = 0.22; $I^{2}$ = 0%) [Fig. 3].

#### 3.2.3 Safety Endpoint

**Fig. 4.**
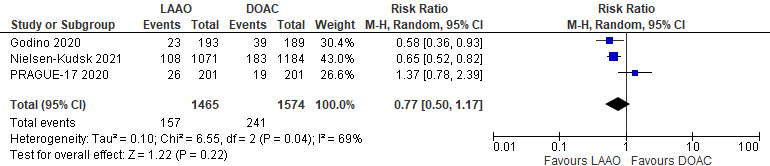
**Forrest plot comparing LAAO to DOAC for the safety endpoint**.

All three studies reported the incidence of bleeding events (both major and minor). A total of 157 events in 1465 patients (10.7%) occurred in the LAAO group, and 241 events in 1574 patients (15.3%) occurred in the DOAC group. There was no statistically significant difference between the two groups in terms of bleeding events (RR 0.77; 95% CI 0.50–1.17; *p* = 0.22) [Fig. 4]. However, moderate heterogeneity among the studies included in the analysis ($I^{2}$ = 69%). Sensitivity analysis involving the removal of each of the studies one at a time demonstrated that PRAGUE-17 influenced the summary risk estimates for bleeding events; After excluding this study, a significant difference favoring LAAO when compared to DOAC was observed (RR: 0.64; 95% CI: 0.52–0.78; *p *< 0.0001), with homogenous findings ($I^{2}$ = 0%) (**Supplementary Fig. 5**).

#### 3.2.4 Procedure/Device Related Complications

A total of 33 complications, including device-related deaths, device embolization, vascular complications, pericardial effusion, and other complications like malposition or leak were reported in the LAAO arm. Overall, the incidence of procedure/device-related complications at follow-up were low (2.2%) and comparable among the different occluder devices used for LAAO procedures.

#### 3.2.5 Publication Bias

On visual assessment, the funnel plot was symmetrical with an equal number of studies on each side of the vertical axis. There was no publication bias demonstrated. Egger’s test for the assessment of publication bias was non-significant (2-tailed *p *> 0.05) (**Supplementary Figs. 6,7,8**).

## 4. Discussion

In this meta-analysis of 3 studies including 3039 patients, we compared the clinical outcomes of LAAO versus DOAC in patients with non-valvular AF. The principal findings of the meta-analysis are as follows: (1) LAAO and DOACs were overall comparable in terms of safety and efficacy (2) there is a marginal benefit to lower incidence of cardiovascular mortality and bleeding events in the LAAO group, which is not statistically significant probably because of the different definitions of composite endpoints for efficacy and safety outcomes differed across studies. To our best knowledge, this is the first head-to-head meta-analysis comparing the clinical outcomes of LAAO and DOACs in patients with high-risk AF, including the results of the recent PRAGUE-17, Godino *et al*. and Nielsen-Kudsk *et al*. [16, 17, 18]. A prior network meta-analysis had explored the efficacy and safety of LAAO and DOACs but was underpowered because of the paucity of data and the limited number of studies present. It demonstrated that LAAO was less efficacious than DOACs in preventing ischemic stroke but performed better than DOACs in avoiding major bleeding events [19]. In addition to comparing LAAO and DOACs directly, our study differs with respect to outcomes as we showed that LAAO was non-inferior to DOAC in preventing ischemic stroke, cardiovascular mortality and bleeding events. Evidence for LAAO came from 2 major trials, PROTECT-AF (Watchman Left Atrial Appendage System for Embolic Protection in Patients with Atrial Fibrillation) and PREVAIL (Prospective Randomized Evaluation of the Watchman Left Atrial Appendage Closure Device in Patients With Atrial Fibrillation Versus Long-Term Warfarin Therapy) where patients were randomized to either WATCHMAN device or warfarin [20, 21]. In the pivotal PROTECT AF trial, the percutaneous closure of the left atrial appendage with the WATCHMAN device met non-inferiority and superiority criterion for the first co- primary efficacy endpoint (composite outcome of stroke, systemic embolism, and cardiovascular death) at one year and four years of follow-up. However, the subsequent PREVAIL trial failed to achieve the pre-specified criteria for non-inferiority, raising some concerns about the overall effectiveness of the procedure, at least in patients eligible for oral anticoagulation. Although studies have evaluated patients that were eligible for oral anticoagulation, in real-world practice, there is a shift towards LAAO in patients with high-risk AF that are contraindicated to anticoagulation or deemed at prohibitive risk of bleeding [22]. As suggested by the current American College of Cardiology/American Heart Association and European Society of Cardiology guidelines [23, 24], patients with high HAS-BLED score and contraindication for long term oral anticoagulation therapy (e.g., patients who have experienced previous major bleedings like intracranial haemorrhage) can be considered as candidates for LAAO (class II B level of evidence). Peri-procedural and device-related complications during follow-up are the two major concerns about left atrial appendage occluders that may affect the overall outcome of this technique. In addition to bleeding, peri-device leaks and device-related embolization are other concerns. However, most studies found that neither peri-device leak <5 mm nor device-related thrombosis of LAAO are associated with stroke. A steady decline in complication rates has been documented since LAAO launched on the market. Complications occurred in 8.7% in PROTECT-AF, 4.6% in the observational study by Godino *et al*. [17], 4% in Amulet Observational Registry and further decreased in the Watchman EWOLUTION registry and PRAGUE-17 to overall complication rates of 2.7%. and 2.1%, consistent with this improving trend [16, 17, 18, 20, 25]. On the other hand, high discontinuation rates of DOACs have been reported in many extensive registry studies, with bleeding as one of the underlying causes [4, 5, 26]. The high DOAC discontinuation rate should, in theory, add to a higher risk of ischemic stroke, but the incidence of ischemic stroke/TIA did not differ significantly between the two arms in our analysis. Data comparing LAAO with DOAC directly are still limited. Several randomized clinical trials comparing LAAO with DOAC has now been commenced, such as the OPTION (Comparison of anticoagulation with LAAO after AF ablation; NCT03795298), OCCLUSION-AF (LAAO versus DOAC for stroke prevention in AF; NCT03642509), CLOSURE-AF (LAAO in patients with AF compared to medical therapy; NCT03463317), CATALYST (Amplatzer Amulet LAAO vs. DOAC; NCT04226547), and CHAMPION-AF (Watchman FLX vs. contemporary oral anticoagulation; NCT04394546) trials, but results will not be available until 2024. Therefore, in conclusion, LAAO has emerged as a promising alternative to oral anticoagulation in patients with high-risk AF. Ongoing randomized trials focusing on unresolved issues, such as LAAO in oral anticoagulation ineligible patients and head-to-head comparison with the gold standard DOACs are likely to provide definitive data and hence contribute to an inevitable growth and expansion in LAAO procedures in the upcoming years.

## 5. Limitations

Several limitations of our study should be acknowledged. First, the definitions of composite endpoints for primary, secondary, and safety outcomes differed across trials. Second, we assessed a pharmacological approach with an interventional strategy, which have primarily different efficacy and safety profiles; therefore, the variability of approaches might be an essential source of distortion in the observed point estimates. Third, the inclusion of observational studies may have led to bias in our pooled estimates due to residual confounding based on the unavailability of PS matching in all studies. That being said, due to the paucity of data, we included observational studies and RCTs. However, it is also found that the pros of including both observational and RCTs in a meta-analysis outweigh the cons [27]. As a consequence of the arguments described above, we believe that including observational studies provides additional evidence and increases the estimate’s precision.

## 6. Conclusions

Among patients with a high risk for ischemic stroke and bleeding, LAAO was non-inferior to DOACs and showed comparable and reassuring efficacy in thromboembolic event prevention. The findings suggest that if a patient with AF is at high risk for stroke and bleeding complications, LAAO is a reasonable alternative to DOAC therapy.

## Supplementary Material

Supplementary material associated with this article can be found, in the online version, at https://doi.org/10.31083/j.rcm2402044.
