# Supplementary material for: Long-Term Efficacy and Safety of Left Atrial Appendage Occlusion (LAAO) vs Direct Oral Anticoagulation (DOAC) in Patients with Atrial Fibrillation: A Systematic Review and Meta-Analysis
Source: Rev Cardiovasc Med. 2023 Feb 2;24(2):44. doi: 10.31083/j.rcm2402044 (PMC11273138; doi:10.31083/j.rcm2402044)
Supplement: Supplementary file 1 [file 2153-8174-24-2-044-s1.zip › Supplementary File.docx]

Supplementary File

**Sections-**

1. **Search strategy**
2. **Quality Assessment**
3. **Sensitivity Analysis**
4. **Publication Bias**

# Section 1 -


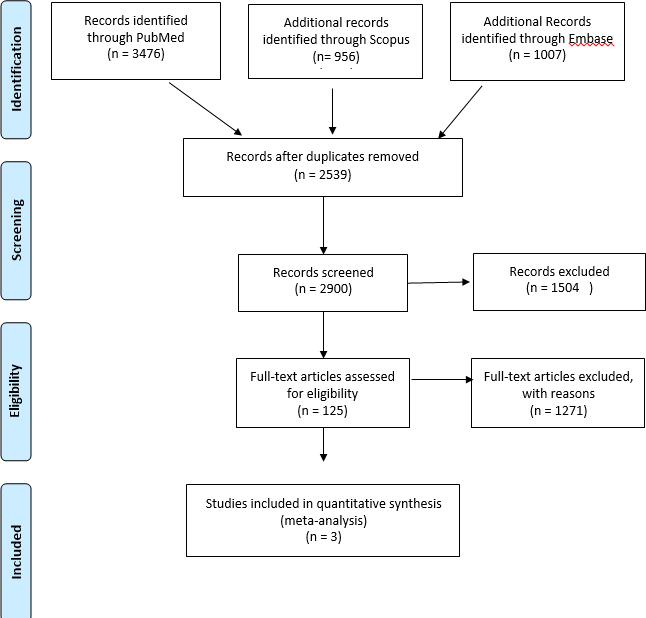


Figure 1

# Section 2

| **Quality Assessment criteria** | **Acceptable(*)** | **Okura21 (-)** | **Jones22 (-)** |
| --- | --- | --- | --- |
| **Representativeness of exposed cohort?** | **Representative of average adult with the disease** | * | * |
| **Selection of the non- exposed cohort?** | **Drawn from same community as exposed cohort** | * | * |
| **Ascertainment of exposure?** | **Secured records** | * | ***** |
| **Demonstration that the outcome of interest was not present at the start of study?** | **Secured records** | * | * |
| **Study controls for age/sex?** | **Yes** | * | * |
| **Study controls for at least 3 additional risk factors?** | **BMI, DM, HTN, Dyslipidemia, CAD, Smoking, CKD, COPD, Atrial fibrillation, COVID-19 features** | * | * |
| **Assessment of outcome?** | **Independent**  **blind assessment, record linkage** | ***** | * |
| **Was follow-up long enough for outcome to occur?** | **Follow-up >1 month** | * | ***** |
| **Adequacy of follow-up of cohorts?** | **Complete follow-up, or subjects lost to follow-up unlikely to introduce bias** | ***** | * |
| **Overall Quality Score (Maximum = 9)** | | **9** | **9** |

**Supplemental Table 1**-Newcastle-Ottawa scale for assessment of quality of included studies – Cohort studies (each asterisk represents if individual criterion within the subsection was fulfilled)


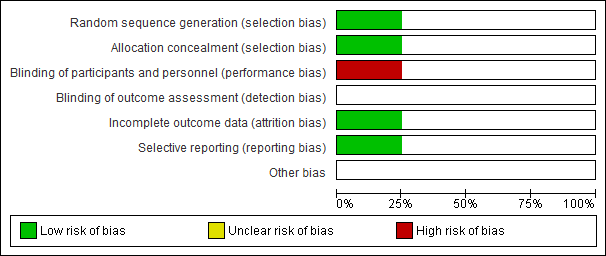


**Supplemental Figure 1-** Cochrane risk of Bias Graph for Randomised controlled trials

**Supplemental Figure 2-** Cochrane risk of Bias Graph for Randomized controlled trials

## Section 3-


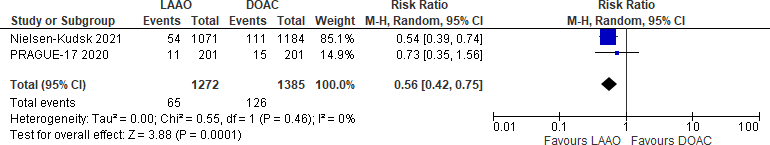


**Supplemental Figure 3**- Sensitivity Analysis Forrest plot for Cardiovascular Mortality


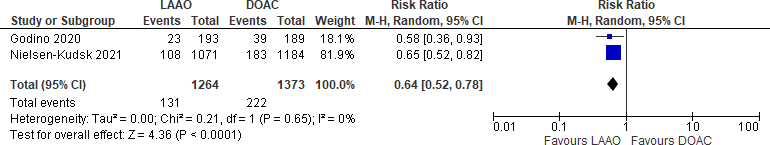


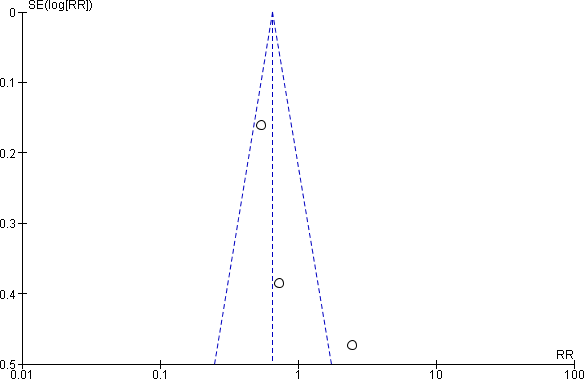
**Supplemental Figure 4-** Sensitivity Analysis Forrest plot for Safety Endpoint Section 4:

## Cardiovascular Mortality


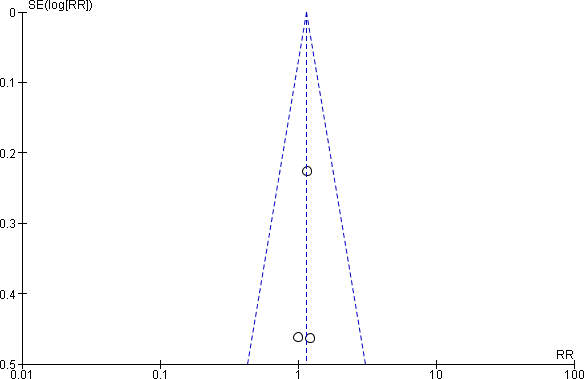


1.
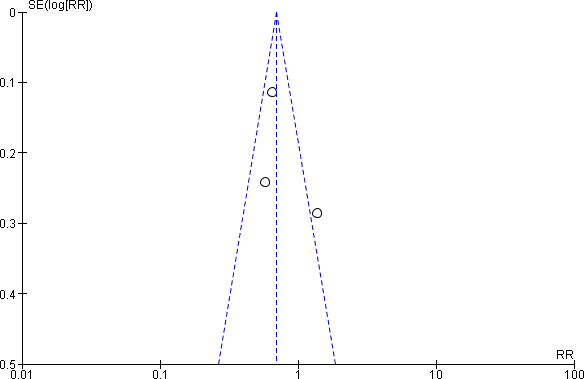
Stroke/TIA

## Safety Endpoint (Major and Minor bleeding)
